# A genome-wide association study implicates the pleiotropic effect of *NMUR2* on asthma and COPD

**DOI:** 10.1038/s41598-022-24766-6

**Published:** 2022-12-21

**Authors:** Ah Ra Do, Jin An, Jinyeon Jo, Woo Jin Kim, Hae Yeon Kang, Sanghun Lee, Dankyu Yoon, You Sook Cho, Ian M. Adcock, Kian Fan Chung, Sungho Won, Tae-Bum Kim

**Affiliations:** 1 Interdisciplinary Program of Bioinformatics, Seoul National University, Seoul, South Korea; 2 Department of Pulmonary, Allergy and Critical Care Medicine, Kyung Hee University Hospital at Gangdong, College of Medicine, Kyung Hee University, Seoul, South Korea; 3 Department of Public Health Sciences, School of Public Health, Seoul National University, Kwanak-ro 1, Kwanak-gu, Seoul, 151-742 Korea; 4 Department of Internal Medicine and Environmental Health Center, Kangwon National University, Chuncheon, South Korea; 5 Department of Internal Medicine, Healthcare Research Institute, Seoul National University Hospital Healthcare System Gangnam Center, Seoul, South Korea; 6 Deptartment of Medical Consilience, Division of Medicine, Graduate School, Dankook University, Yongin, South Korea; 7 Division of Allergy and Respiratory Disease Research, Department of Chronic Disease Convergence Research, National Institute of Health, Korea Disease Control and Prevention Agency, Cheongju, Korea; 8 Department of Internal Medicine, Division of Allergy and Clinical Immunology, Asan Medical Center, Ulsan University School of Medicine, Seoul, South Korea; 9 Experimental Studies, Airways Disease Section, National Heart and Lung Institute, Imperial College London, London, UK; 10 Institute of Health and Environment, Seoul National University, Seoul, South Korea; 11 RexSoft Inc., Seoul, Korea; 12 Department of Allergy and Clinical Immunology, Asan Medical Center, University of Ulsan College of Medicine, 86 Asanbyeongwon-gil, Songpa-gu, Seoul, 138-736 Korea

**Keywords:** Genetics, Biomarkers

## Abstract

Asthma and chronic obstructive pulmonary disease (COPD) are two distinct diseases that are associated with chronic inflammation. They share common features in terms of their advanced stages and genetic factors. This study aimed to identify novel genes underlying both asthma and COPD using genome-wide association study (GWAS) to differentiate between the two diseases. We performed a GWAS of asthma and COPD in 7828 Koreans from three hospitals. In addition, we investigated genetic correlations. The UK Biobank dataset was used for the replication studies. We found that rs2961757, located near neuromedin U receptor 2 (*NMUR2*) on chromosome 5, was genome-wide significant (${\upbeta }_{\mathrm{Asthma}-\mathrm{COPD}}$ = 0.44, *P*-value_Asthma-COPD_ = 3.41 × 10^−8^), and significant results were replicated with the UK Biobank data (${\upbeta }_{\mathrm{Asthma}-\mathrm{COPD}}$ = 0.04, *P*-value_Asthma-COPD_ = 0.0431). A positive genetic correlation was observed between asthma and COPD (39.8% in the Korean dataset and 49.8% in the UK Biobank dataset). In this study, 40–45% of the genetic effects were common to asthma and COPD. Moreover, *NMUR2* increases the risk of asthma development and suppresses COPD development. This indicates that *NMUR2* allows for better differentiation of both diseases, which can facilitate tailored medical therapy.

## Introduction

Asthma and chronic obstructive pulmonary disease (COPD) are characterized by chronic inflammation and remodeling of the airways^1^. Despite some clinical and physiological similarities, both diseases exhibit distinct features related to the underlying inflammatory patterns, mechanisms involved in airway obstruction, and prognosis^2,3^. These differences are reflected in the treatment approaches used for asthma and COPD^4^. Given the critical importance of choosing the optimal treatment for both diseases, a clear distinction between asthma and COPD is desirable.

Asthma and COPD are often difficult to distinguish clearly in clinical practice because of their common characteristic traits. Some patients with clinical features of both asthma and COPD are referred to as asthma-COPD overlap (ACO)^5^. It has been hypothesized that there may be a shared and underlying genetic predisposition in both diseases. The largest recent genome-wide association study (GWAS) identified eight signals for ACO and suggested a spectrum of shared genetic impacts on asthma, COPD, or lung function^6^.

An association of some genes with two different diseases is known as pleiotropy. To the best of our knowledge, no GWASs has identified single nucleotide polymorphisms (SNPs) with pleiotropic effects on asthma and COPD comparing asthma, COPD, and control subjects concurrently^7^. This study aimed to identify novel genes underlying both asthma and COPD using GWAS to differentiate between the two diseases. We first performed GWAS for asthma, COPD, and healthy controls in South Korea, followed by a replication study, and then estimated the heritability and genetic correlations between asthma and COPD.

## Methods

### Study population, design, data summary, and quality control

#### Discovery data

A total of 7828 participants were recruited from three hospitals: Asan Medical Center (AMC), Kang-Won National University Hospital (KNUH), and Seoul National University Hospital Healthcare system Gangnam Center (SNUH). The subjects included 1391 patients with asthma, 1091 patients with COPD, and 5346 controls. Of the 1391 asthmatic patients, 1295 and 96 were from AMC and SNUH, respectively. The asthmatic patients from AMC were aged > 18 years with airway hyperresponsiveness, as indicated by a 20% reduction in forced expiratory volume in 1 s (FEV1) with a methacholine dose of 16 mg/mL through a provocation test, or airway reversibility in FEV1 > 12% (and at least 200 mL) after inhalation of a short-acting β-agonist. For the SNUH dataset, 96 asthmatic patients who provided a “yes” response to either of the following questions in health checkup questionnaire were included in this study: “Have you ever been diagnosed with asthma?” “Have you ever taken asthma medication at least once?”.

Of the 1091 patients with COPD, 816 and 275 were from KNUH and SNUH, respectively. COPD patients were diagnosed if (1) they were ≥ 40 years of age, (2) they had a spirometry-confirmed airflow limitation (FEV1/forced vital capacity (FVC) of < 0.7), and (3) they were not diagnosed with asthma. ACO cases were identified if asthmatic patients were older than 40 years and had FEV1/FVC < 0.7, and were excluded from our analyses to clearly distinguish asthma from COPD.

Of the 5346 subjects in the control group, 5302 and 44 were from SNUH and KNUH, respectively. Controls subjects were not diagnosed with asthma or COPD. All patients provided written informed consent, and the study was approved by the Institutional Review Boards (IRB) at the AMC (2019-1676), KNUH (2015-08-010-021), and SNUH (H-2005-209-1127).

#### Replication data

We used UK Biobank (UKB) data for the replication study. UKB is a large long-term biobank study in the United Kingdom that aims to investigate genetic predispositions to diseases and the contributions of environmental exposure^8^. We considered the British, any other white background, and Irish people. Subjects were excluded if they (1) did not have information of height, (2) did not have two or more lung function test results, and (3) did not pass the American Thoracic Society (ATS) quality control (QC). The asthmatics for UKB were determined patients who have (1) a record for the 'age asthma diagnosed' item or (2) ‘1111 (asthma)’ for 'self-reported non-cancer illness code’. Patients with COPD were included based on (1) FEV1/FVC ratio < 0.7; (2) a recode for ‘age COPD diagnosed by a doctor’; or (3) ‘1112 (COPD)’ for ‘self-reported non-cancer illness code’.

#### Genotyping, imputation, and quality controls

**Figure 1:**
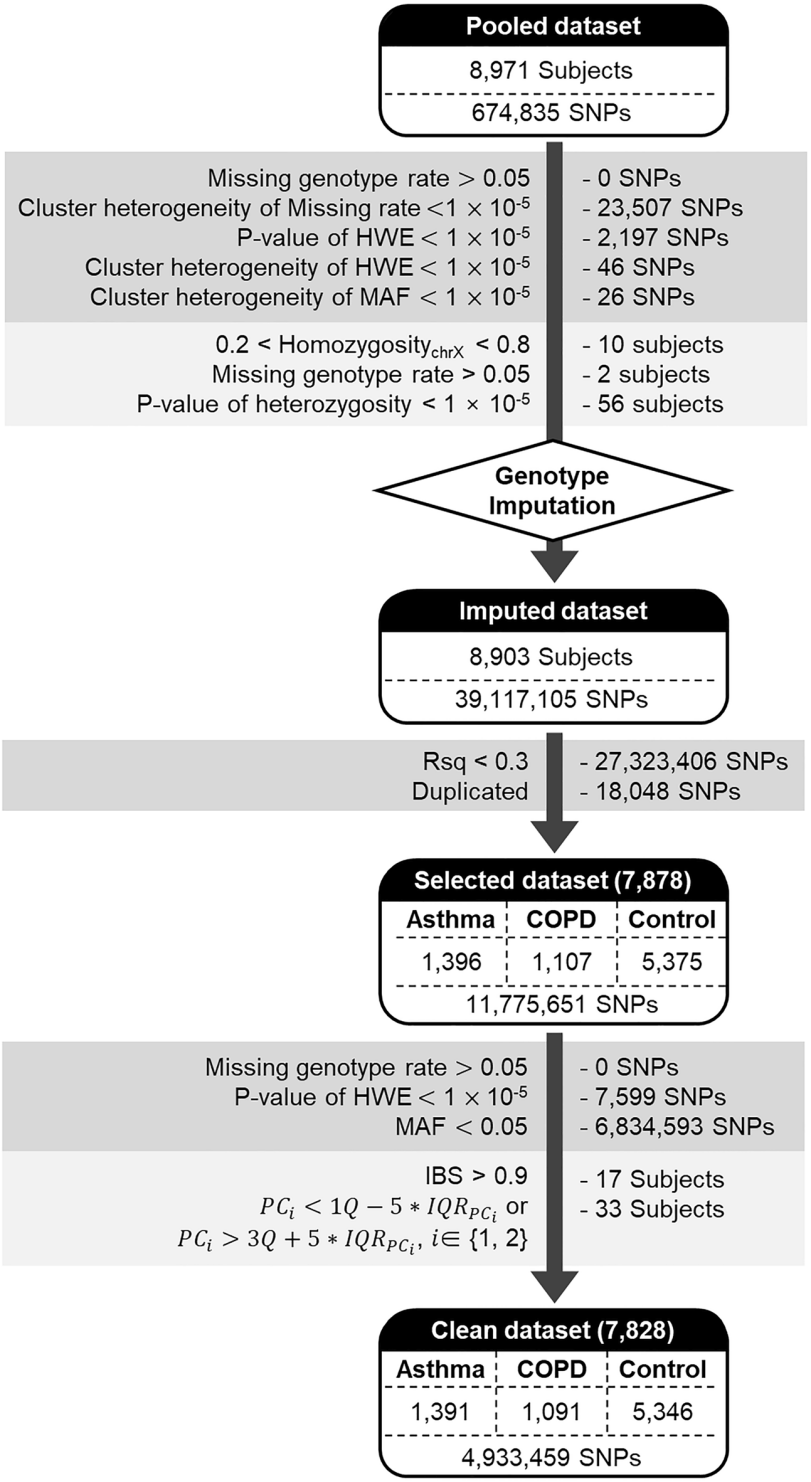
Workflow for the asthma-COPD GWAS using the Korean dataset. Multiple standard quality controls were performed for the asthma, COPD, and control groups to exclude outlier SNPs and subjects.

Discovery subjects were genotyped using the Korea Biobank Array (referred to as Korean Chip)^9^. The variants are called Best Practices Steps^10^. To minimize batch effects, genotype calling and upstream quality control were conducted using the K-medoid clustering-based method^11^. QC of the SNPs and subjects was performed using PLINK^12^ and ONETOOL^13^. SNPs were excluded if any of the following conditions were satisfied: (1) genotype call rates of < 95% and (2) *p*-value for the Hardy–Weinberg equilibrium (HWE) test of < $1\times {10}^{-5}$.^14^ Subjects were excluded based on the following criteria: (1) 0.2 < X chromosome homozygosity < 0.8; (2) genotype call rate < 95%; and (3) heterozygosity rates of SNPs outside the average heterozygosity rate ± 3 standard deviations. QC was applied to each cluster and the pooled samples (Fig. 1). In addition, we compared the missing rates, HWE, and minor allele frequencies (MAFs) of the clusters using Pearson’s chi-square test. SNPs with *P-values* less than 1 × 10^−5^ were discarded. After the QC process, 8,903 subjects and 649,085 SNPs remained and were utilized for imputation.

Imputation was performed using the Michigan imputation server (https://imputationserver.sph.umich.edu). We used the Haplotype Reference Consortium release v1.1 and only considered non-European or mixed populations^15^. Pre-phasing and imputation were conducted using Eagle’s V 2.4^16^ and Minimac4^17^, respectively. Subsequently, some imputed SNPs were removed for the following: (1) Rsq < 0.3, (2) duplicates, (3) missing genotype rates of > 0.05, (4) *P*-values for HWE of < 1 × 10^−5^, or (5) MAFs < 0.05. In addition, the subjects with an identity-by-descent (IBS) of > 0.9 and a principal component (PC) score outside the 5 × IQR_PC_ were removed. Finally, 4,933,459 SNPs from 1391 asthmatic patients, 1091 COPD patients, and 5,346 controls were used for our analyses.

### Genome-wide association studies

To determine the genomic differences between the three groups, we performed multinomial logistic regression using the 'multinom' function in the 'nnet' package for R and the generalized logit link function for the SNPs and Rex (Version 3.0.3)^18^. Age, sex, and 20 PC scores were included as covariates. We fitted the full and reduced models, and the likelihood ratio test (LRT), which follows a chi-squared distribution with two degrees of freedom, and it was used to identify genome-wide significant SNPs. We also added smoking status as a covariate for the top 10 significant SNP to determine the effects caused by smoking history.

Full model:

$$\begin{aligned}\mathrm{log}\frac{P\left(Asthma\right)}{P\left(Control\right)} &={\beta }_{0}^{Asthma}+{\beta }_{1}^{Asthma}{\mathrm{SNP}}_{\mathrm{i}}+{\beta }_{2}^{Asthma}\mathrm{Age}+{\beta }_{3}^{Asthma}\mathrm{Sex}+{\beta }_{4}^{Asthma}\mathrm{PC}1+\cdots +{\beta }_{23}^{Asthma}\mathrm{PC}20\\ \mathrm{log}\frac{P(COPD)}{P(Control)}&={\beta }_{0}^{COPD}+{\beta }_{1}^{COPD}{\mathrm{SNP}}_{\mathrm{i}}+{\beta }_{2}^{COPD}\mathrm{Age}+{\beta }_{3}^{COPD}\mathrm{Sex}+{\beta }_{4}^{COPD}\mathrm{PC}1+\cdots +{\beta }_{23}^{COPD}\mathrm{PC}20\end{aligned}$$

Reduced model:

$$\begin{aligned}\mathrm{log}\frac{P(Asthma)}{P(Control)} &={\beta }_{0}^{Asthm}+{\beta }_{2}^{Asthma}\mathrm{Age}+{\beta }_{3}^{Asthma}\mathrm{Sex}+{\beta }_{4}^{Asthma}\mathrm{PC}1+\dots +{\beta }_{23}^{Asthma}\mathrm{PC}20 \\ \mathrm{log}\frac{P(COPD)}{P(Control)}&={\beta }_{0}^{COPD}+{\beta }_{2}^{COPD}\mathrm{Age}+{\beta }_{3}^{COPD}\mathrm{Sex}+{\beta }_{4}^{COPD}\mathrm{PC}1+\dots +{\beta }_{23}^{COPD}\mathrm{PC}20\end{aligned}$$

The null hypothesis for the proposed chi-squared test was ${\beta }_{1}^{Asthma}={\beta }_{1}^{COPD}=0$, and if both ${\beta }_{1}^{Asthma}$ and ${\beta }_{1}^{COPD}$ were significantly nonzero, the SNP was assumed to have a pleiotropic effect. We also used the Wald test to test for significant differences between patients with asthma (or COPD) and the controls.

### Genetic heritability and correlation assessment

We estimated the SNP heritability of asthma and COPD and their genetic correlations using both discovery and replication data. For the discovery data, SNPs were pruned with PLINK using the default option. We applied the bivariate genomic restricted maximum likelihood (GREML) of GCTA64 as the default option to estimate the genetic correlation between asthma and COPD and heritability. The prevalence of asthma and COPD were set to be 0.04 and 0.14, respectively, to remove the ascertainment sampling bias^19,20^. For the UKB dataset, we used the linkage disequilibrium score regression (LDSC) method with the default option^21,22^ because the dataset was too large to process with GCTA64.

### Hi-C analysis

To evaluate the associations between significant SNP and target genes, Hi-C data from the fetal lung fibroblast cells using the HUGIn^23^ web browser was obtained. Hi-C measures the frequency of physical binding of DNA sculptures in three-dimensional space and shows the characteristics of genes that are close to each other in chromosome topological structure.

### RNA expression association test

We determined whether the mRNA expression levels of genes annotated by genome-wide significant SNPs differed significantly across the diseases. Gene expression datasets were obtained from the GEO and U-BIOPRED^24^ databases. We considered five datasets from the GEO database: GSE104472, GSE23552, and GSE59019 with asthmatic patients and controls and COPD patients and controls; GSE29133 with asthmatic patients and COPD patients; and GSE112250. A significant difference was detected using the 'limma' method in the R package^25^. In the U-BIOPRED data, 446 patients with moderate to severe asthma and 91 controls were assessed for *NMUR2* expression using bronchial biopsy.

### Ethical approval

This study was performed in line with the principles of the Declaration of Helsinki. The study protocol was approved by the Institutional Review Boards (IRB) at the Asan Medical Center (2019-1676), Kang-Won National University Hospital (2015-08-010-021), and Seoul National University Gangnam Center Seoul National University Hospital (H-2005-209-1127).

## Results

### Subjects characteristics

The demographic characteristics of the 1391 asthmatic patients, 1091 COPD patients, and 5346 controls are shown in Table 1. The average age of the asthmatic patients, COPD patients, and controls was 50.3, 66.6 and 49.7, respectively. The proportion of females was higher in the asthma group than in the COPD and control groups (55.3% vs. 11.4% and 44.1%, respectively). The average FEV1 for patients with COPD was 66.0%, and those for asthmatic patient and controls were 78.8% and 101.7%, respectively. The pre-bronchodilator FEV1/FVC of COPD patients was the lowest at 54.8%, and those of asthmatic patients and controls were 70.7% and 81.6%, respectively.

**Table 1:** Demographic characteristics of the discovery dataset.

Variable	Asthma	COPD	Control	Total	*P-*value
	(N = 1391)	(N = 1091)	(N = 5346)	(N = 7828)	
Age (yr)	50.3 ± 15.4	66.6 ± 9.9	49.7 ± 10.4	52.2 ± 12.8	< 0.001
Female sex, n (%)	769 (55.3)	124 (11.4)	2355 (44.1)	3248 (41.5)	< 0.001
Height (cm)	162.9 ± 9.2	165.1 ± 8.4	166.4 ± 8.1	165.7 ± 8.4	< 0.001
BMI	24.3 ± 3.6	23.2 ± 3.1	23.1 ± 3.1	23.3 ± 3.2	< 0.001
Pre-BD FEV1 pred (%)	78.8 ± 19.9	66.0 ± 22.7	101.7 ± 12.2	92.9 ± 20.8	< 0.001
Pre-BD FEV1/FVC (%)	70.7 ± 12.1	54.8 ± 12.5	81.6 ± 5.7	76.0 ± 12.6	< 0.001

The mean ± SD values are shown for each cell. BMI, body mass index; FEV1, forced expiratory volume in 1 s; BD, bronchodilator; FVC, forced vital capacity. ANOVA and X^2^ tests were performed for quantitative and qualitative variables, respectively.

### Genome-wide association between COPD, Asthma, and Control in the Korean dataset

**Figure 2:**
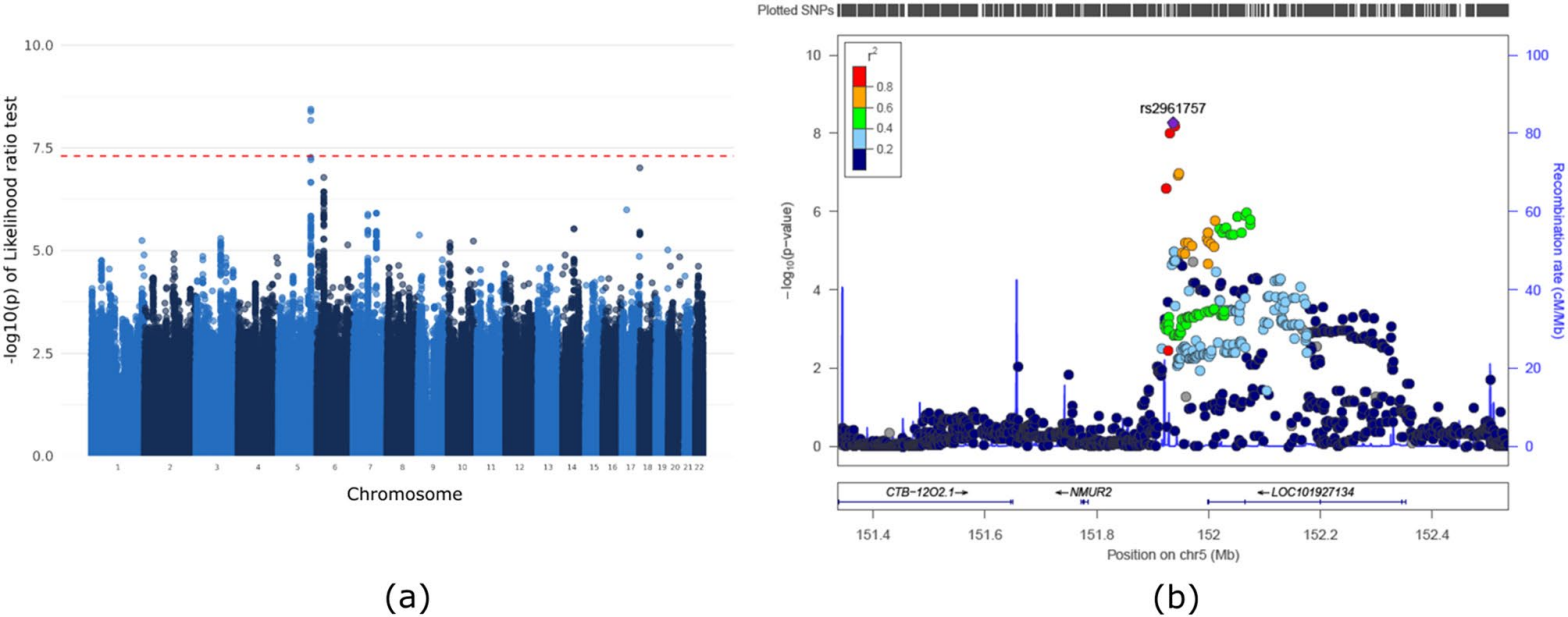
GWAS results for the Korean dataset. (**a**) Manhattan plot of the likelihood ratio test of the SNPs from chromosomes 1 to 22. (**b**) Expanded Manhattan plot of the 800 kb region shows both genotyped and imputed SNPs. rs2961757, which was the most significant SNP, and is indicated in purple, and other SNPs are colored according to their r^2^ value relative to that of rs2961757. *NMUR2* and *LOC101927134* genes are located in this range.

**Figure 3:**
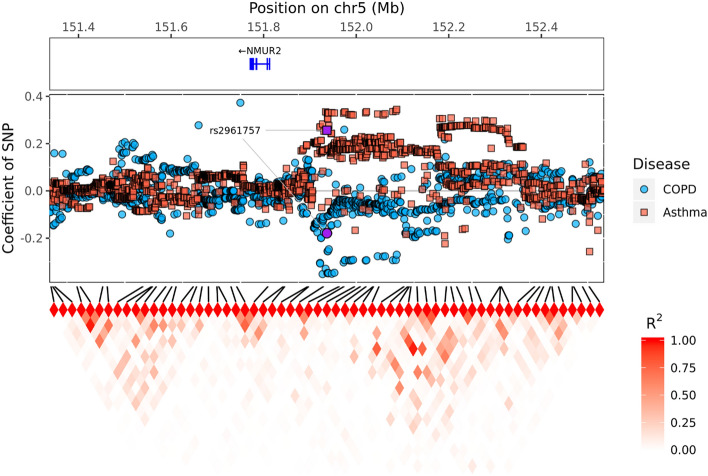
SNP coefficients for Asthma and COPD disease in the target region. The top box shows the located coding gene in the target region. The middle box represents the coefficients of SNPs. The red and blue squares represent the coefficients of SNPs for the asthmatic patients and controls and the COPD patients and controls, respectively. The tile graph at the bottom shows the correlation between the genotyped SNPs, and the amount of correlation is shown by the darkness of the red color.

We assumed that SNPs can affect COPD and/or asthma and conducted a GWAS using multinomial logistic regression. The null hypothesis, which assumed that SNPs did not have any effect on either disease, was tested, and we found significant SNPs on chromosome 5. Figure S1 shows a multidimensional scaling (MDS) plot based on our discovery dataset and the 1000 Genomes Phase 3 dataset and provides evidence of no population stratification. Figure S2 shows a quantile–quantile (QQ) plot for LRT with two degrees of freedom. Inflation was not observed (the genomic inflation factor ($\uplambda$) was 1.048 for all SNPs and 1.027 for pruned SNPs). In Fig. 2, Manhattan and regional plots indicate a genome-wide significant region. rs2961757 is located near *NMUR2* on chromosome 5. In addition, in the first plot of Fig. S3, the genotyped SNP is indicated by a red dot in the Manhattan plot. The regional plot was produced using only genotyped SNP. The Miami plot of the results is presented in Fig. S4. Table 2 lists the top ten most significant SNPs. Minor allele frequencies in the disease group are shown in Table S1. If several genome-wide significant SNPs were present in the same linkage disequilibrium (LD) block, only the most significant SNPs were included. The top ten SNPs were compared using the minor allele frequency of the SNPs in the Korean reference dataset (Kref), which consisted of data from 66,407 subjects. The rs2961757 is genome-wide significant for the LRT (*P*-value_LRT_ = 3.61 × 10^−9^), and the Wald test outcomes for the two different groups were significant (${\upbeta }_{\mathrm{COPD}-\mathrm{Con}}$ = -0.18, *P*-value_COPD-Con_ = 0.0105; ${\upbeta }_{\mathrm{Asthma}-\mathrm{Con}}$ = 0.26, *P*-value_Asthma-Con_ = 1.18 × 10^−7^; ${\upbeta }_{\mathrm{Asthma}-\mathrm{COPD}}$ = 0.44, *P*-value_Asthma-COPD_ = 3.41 × 10^−8^). Figure S5 shows that the frequency of the minor allele T in asthma is higher than that in COPD. Furthermore, in Fig. 3, we show that asthma and COPD have positive and negative associations when compared to controls in certain regions close to the *NMUR2* gene. These estimates indicate that rs2961757 increases the risk of asthma but decreases the risk of COPD. The largest difference was found between asthmatic and COPD patients. We further adjusted for the effect of smoking on the top 10 SNPs. The results are presented in Table S2, which shows that rs2961757 remained genome-wide significant (*P*-value_LRT =_ $1.47\times {10}^{-8}$).

**Table 2:** The top 10 most significant SNPs.

Chr	SNP	Position	Alleles	LR	*P*-value*	$${\beta }^{**}$$	*P*-value**	$${\beta }^{***}$$	*P*-value***	$${\beta }^{****}$$	*P*-value****	Gene
5	rs2961757	151,936,718	T/G	38.88	3.61 × 10^−9^	− 0.18	0.01	0.26	$1.18\times {10}^{-7}$	0.44	3.41 × 10^−8^	*NMUR2, LINC01470*
18	rs9303897	2,306,842	G/A	32.29	9.74 × 10^−8^	0.36	8.11 × 10^−8^	− 0.06	0.23	− 0.42	7.19 × 10^−8^	*LINC00470, METTL4*
6	rs1770	32,627,833	G/A	31.21	1.67 × 10^−7^	− 0.03	0.67	0.31	$3.74\times {10}^{-8}$	0.34	7.14 × 10^−5^	*HLA-DQB1-AS1*
6	rs10947233	32,124,424	T/G	29.13	4.74 × 10^−7^	− 0.47	$1.16\times {10}^{-7}$	− 0.03	0.65	0.45	3.27 × 10^−6^	*PPT2-EGFL8*
17	rs146362423	21,488,273	C/G	27.58	1.03 × 10^−6^	− 0.53	$1.3\times {10}^{-6}$	0.07	0.36	0.60	1.12 × 10^−6^	*C17orf51, UBBP4*
7	rs1568778	109,250,932	T/C	27.20	1.24 × 10^−6^	0.08	0.21	− 0.24	$1.83\times {10}^{-6}$	− 0.32	2.14 × 10^−5^	*C7orf66, EIF3IP1*
7	rs115881004	68,928,894	T/C	27.08	1.32 × 10^−6^	0.35	$5.29\times {10}^{-5}$	− 0.19	0.01	− 0.54	1.77 × 10^−7^	*LOC102723427, LOC100507468*
6	rs114968045	32,666,960	C/T	25.76	2.54 × 10^−6^	− 0.19	0.02	0.24	$3.35\times {10}^{-5}$	0.43	8.59 × 10^−7^	*HLA-DQB1, HLA-DQA2*
14	rs11623972	73,381,520	G/A	25.45	2.98 × 10^−6^	− 0.32	$6.45\times {10}^{-7}$	− 0.05	0.27	0.27	2.81 × 10^−4^	*DPF3, DCAF4*
9	rs10758715	5,882,401	C/T	24.75	4.22 × 10^−6^	− 0.11	0.11	− 0.25	$1.51\times {10}^{-6}$	− 0.14	7.33 × 10^−2^	*ERMP1, MLANA*

Chr, chromosome; LR, likelihood ratio; *, LR test; **, COPD-control; ***, asthma-control; ****, asthma-COPD.

### Replication with UK Biobank dataset

The data for 13,475 patients with asthma, 7822 patients with COPD, and 109,871 controls were included in the replication analysis (Table S3). We performed replication analysis using the UKB dataset (Table S4). Neither the imputed nor genotyped rs2761757 was available. Alternatively, rs12655008, which had the largest correlation (r^2^ = 0.51), was considered. For the discovery analysis, the largest difference was found between asthmatic and COPD patients, and it was significantly associated with the same direction in the replication analysis (*P*-value_Asthma-COPD_ = 0.0431, Fig. S6).

### Hi-C analysis

The relationship between the rs2961757 and *NMUR2* genes in the fetal lung fibroblast cells was identified in the Hi-C browser on chromosome 5 (Fig. S7). The *NMUR2* gene and rs2961757 were observed to be significantly closer than other nearby genes at the false discovery rate (FDR) level of 0.05.

### RNA expression association test

**Figure 4:**
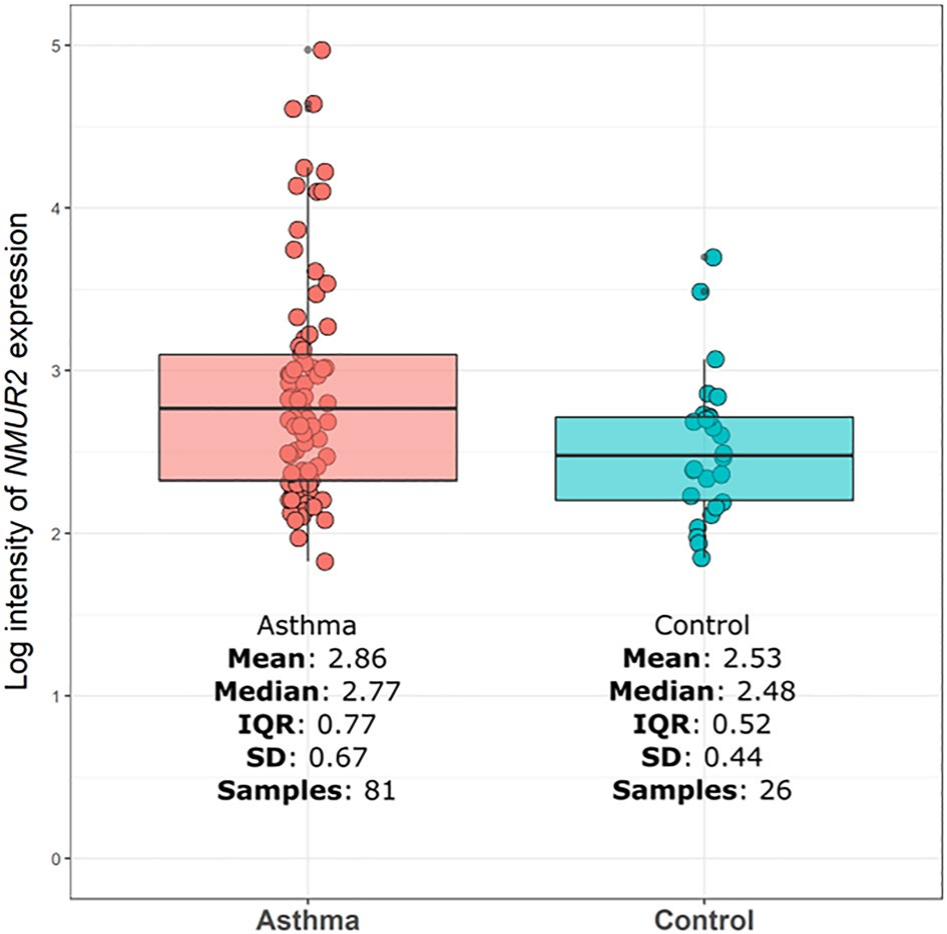
Analysis of RNA expression of *NMUR2* in asthma using U-BIOPRED. *NMUR2* expression was measured in the bronchial tissue of 81 asthma samples and 26 controls. Box plot shows log intensity of the *NMUR2* in asthma and controls.

*NMUR2* is most closely located near the genome-wide significant SNP rs2961757. The mRNA expression of *NMUR2* in asthmatic patients, COPD patients, and controls was compared (Table 3, Fig. 4). Compared with those observed for the five GEO datasets and the U-BIOPRED dataset, the expression of the *NMUR2* gene was increased in asthmatic patients and decreased in COPD patients compared with the controls. Furthermore, asthmatic patients showed greater *NMUR2* gene expression than COPD patients.

**Table 3:** Differentially expressed gene analysis using GEO datasets.

Case-Con	Dataset	Country	ID	Gene	$\beta$	*P*-value	Tissue	Covariates	# of Case	# of Con
Asthma-Con	U-BIOPRED	UK		NMUR2		0.0049	Bronchial biopsy		446	92
Asthma-Con	GSE104472	USA	cg07429087	NMUR2	2.28	0.0233	Bronchial epithelia	Sex and age	12	12
Asthma-Con	GSE23552	USA	2,882,325	NMUR2	2.45	0.0980	Nasosinus tissue		22	17
Asthma-Con	GSE59019	USA	11729498_at	NMUR2	0.11	0.0224	PBMC	Sex and stim	26	12
COPD-Con	GSE29133	Japan	224088_at	NMUR2	− 5.18	0.0630	Lung tissues	Sex and age	3	3
Asthma-COPD	GSE112260	Poland	17,001,981	NMUR2	0.70	0.0876	Isolated alveolar macrophages		8	4

Con, control; PBMC, peripheral blood mononuclear cells; #, number.

### Genome-wide genetic correlation and SNP-based heritability

We estimated the heritability of asthma and COPD and their genetic correlation with GCTA64 in the Korean dataset and with LDSC in the UKB dataset. The heritability of asthma and COPD was 39.8% and 49.8%, with a prevalence of 0.04 and 0.14, respectively, in the Korean population. The positive correlation was 0.4068 for the discovery data. For the UKB data, the heritability was 5.5% for asthma and 6.1% for COPD, and the correlation was 0.4547 (Table 4).

**Table 4:** Heritability and genetic correlation of asthma and COPD disease.

Dataset	${h}_{g0}^{2}$	*P*-value (${\mathrm{h}}_{g0}^{2}$)	${h}_{g1}^{2}$	*P*-value (${\mathrm{h}}_{g1}^{2}$)	${\sigma }_{g0}^{2}$	${\sigma }_{g1}^{2}$	${\sigma }_{g0}{\sigma }_{g1}{\rho }_{g}$	${\rho }_{g}$	s.e.$({\rho }_{g})$	*P*-value (${\rho }_{g}$)	${\rho }_{e}$	s.e. (${\rho }_{e}$)
Korean	0.40	3.83 × 10^−8^	0.50	7.00 × 10^−5^	0.04	0.02	0.01	0.41	0.19	7.05 × 10^−3^	− 0.03	0.004
UKBB	0.06	1.77 × 10^−15^	0.06	2.04 × 10^−27^			0.03	0.45	0.06	1.63 × 10^−15^		

$${g}_{0}$$: Asthma, $${g}_{1}$$: COPD.

## Discussion

This is the first study to conduct a GWAS for asthma, COPD, and controls to determine the common genetic polymorphisms contributing to susceptibility to asthma and COPD. We focused on identifying SNPs that jointly affected asthma and COPD. The genetic correlation between asthma and COPD was 41%. It is reasonable to assume that the same SNP can affect both diseases. We used a multinomial approach and identified a novel SNP rs2961757 on chromosome 5, which is located near NMUR2, as a potentially common gene with opposing effects in patients with asthma and COPD. The mRNA expression of *NMUR2* in the U-BIOPRED and GEO datasets also increased in patients with asthma and decreased in those with COPD. Additionally, a substantial positive genetic correlation was observed between asthma and COPD together with similar heritability estimates.

This study contributes to the understanding of shared genetic factors in asthma and COPD based on a GWAS comparing asthma, COPD, and control subjects. We discovered that *NMUR2* is a common gene involved in the development of asthma and COPD, with increased and decreased expression in asthma and COPD, respectively. Additionally, the mRNA expression of *NMUR2* in bronchial and lung tissues in the U-BIOPRED and GEO datasets showed a trend that was consistent with our discovery results. This finding led to the speculation that *NMUR2* may be a novel common gene that increases the risk of asthma but decreases the risk of COPD. Another important question posed in the present study was the extent to which common genes play roles in asthma and COPD. Interestingly, the genetic correlation was 41% between asthma and COPD in our study, which is consistent with the results of the UKB. This could mean that some shared genetic factors mediate the unexpected relationship between asthma and COPD.

Neuromedin U (*NMU*) is a neuropeptide associated with several physiological activities, including weight gain, metabolism, and smooth muscle contraction^26^. Recently, *NMU* has been shown to contribute to inflammation in both innate and adaptive immune responses, which enhances type 2 innate lymphoid cell (ILC2)-driven allergic lung inflammation, leading to eosinophilic inflammation^27–29^. With regard to the signaling pathway, *NMU* activates extracellular signal-regulated kinase phosphorylation and regulates innate type 2 cytokines downstream of the Ca^2+/^calcineurin/NFAT cascade, leading to the expression of the type 2 cytokine genes *IL-5, IL-13* and amphiregulin in *ILC2s*^30^. *NMUR2* is a G protein-coupled receptor recognized as one of the main receptors for *NMU* in the central nervous system and peripheral tissues such as the lungs and testis^26,31–33^. *NMUR2* may mediate the effects of *NMU* in the lungs, leading to type 2 cytokine responses and eosinophilic airway inflammation, which are involved in the development of asthma. Since the nature of airway inflammation in asthma and COPD differs, primarily eosinophilic in asthma and neutrophilic in COPD^34^, the expression of *NMUR2* may be opposing in COPD and asthma considering the effects of *NMUR2* in airway inflammation.

Differentiating between asthma and COPD is important in the treatment approach, because of the discrepancy in the pathophysiology of the two diseases. It is often challenging for physicians to distinguish between diseases during diagnosis or to determine their characteristics because of overlaps between the characteristics of asthma and COPD. The component of chronic irreversible airflow obstruction in patients with long-standing asthma may make diagnosis difficult because the symptoms may be similar to those of COPD^35^. From this perspective, *NMUR2* might be a genetic marker for the differentiation of patients with asthma and COPD. Further functional studies are essential to confirm its potential ability of differentiate between asthma and COPD.

Our study has several limitations. First, controls were selected to exclude subjects with asthma, COPD, or other respiratory diseases, using a standardized questionnaire. Therefore, the selection of controls was potentially vulnerable to misclassification bias owing to the inclusion of undiagnosed cases. Second, patients with asthma may have heterogenous phenotypes. Asthma was ascertained based on the provocation test and bronchodilator response in AMC, but not in SNUH or UKB. The lack of parameters required for phenotyping may be a limitation. Third, asthma and COPD are heterogeneous diseases with gene-environment interactions, and environmental exposure has not been fully assessed. Finally, no post-GWAS functional analyses provided biological insights into the shared genes associated with asthma and COPD. Further functional biological studies related to the role of *NMUR2* are needed to identify individuals at risk for asthma and COPD.

In conclusion, we demonstrated that the SNP rs2961757 of *NMUR2* is the most common significant SNP in patients with asthma and COPD, and it has potentially opposing genetic effects. This finding may have important implications for understanding the common genetic risk factors for the development of these two diseases for the first time. Further studies should focus on the function of *NMUR2* and its clinical usefulness, including asthma treatment strategies beyond genetic profiling.

## Supplementary Information

Supplementary Information.

## Data Availability

For access to data, please contact Tae-Bum Kim and Sungho Won.

## Abbreviations

COPD: Chronic obstructive pulmonary disease
GWAS: Genome-wide association study
SNP: Single nucleotide polymorphism
FEV1: Forced expiratory volume in 1 s
FVC: Forced vital capacity
ACO: Asthma-COPD overlap
UKB: UK Biobank
NMUR2: Neuromedin U receptor 2
HWE: Hardy–Weinberg equilibrium
MAF: Minor allele frequency
PC: Principal component
LD: Linkage disequilibrium
NMU: Neuromedin U
