# Supplementary material for: A genome-wide association study implicates the pleiotropic effect of NMUR2 on asthma and COPD
Source: Sci Rep. 2022 Dec 21;12:22073. doi: 10.1038/s41598-022-24766-6 (PMC9772307; doi:10.1038/s41598-022-24766-6)
Supplement: Supplementary file 1 — Supplementary Information. [file 41598_2022_24766_MOESM1_ESM.docx]

Table S1. SNP information

| **Chr** | **SNP** | **Position** | **Alt/**  **Ref** | **HWE** | **Location** | **Rsq** | **Genotype** | $\boldsymbol{MA}\boldsymbol{F}_{\boldsymbol{All}}$ | $\boldsymbol{MA}\boldsymbol{F}_{\boldsymbol{Asthma}}$ | $\boldsymbol{MA}\boldsymbol{F}_{\boldsymbol{COPD}}$ | $\boldsymbol{MA}\boldsymbol{F}_{\boldsymbol{Con}}$ | $\boldsymbol{MA}\boldsymbol{F}_{\boldsymbol{Kref}}$ | $\boldsymbol{MA}\boldsymbol{F}_{\boldsymbol{1000}\boldsymbol{G}}$ |
| --- | --- | --- | --- | --- | --- | --- | --- | --- | --- | --- | --- | --- | --- |
| 5 | rs2961757 | 151936718 | T/G | 0.418 | intergenic | 0.91 | Imputed | 0.24 | 0.28 | 0.21 | 0.23 | 0.24 | 0.58 |
| 18 | rs9303897 | 2306842 | G/A | 0.001 | intergenic | 0.99 | Genotyped | 0.24 | 0.22 | 0.29 | 0.23 | 0.25 | 0.3648 |
| 6 | rs1770 | 32627833 | G/A | 0.476 | ncRNA  _exonic | 0.60 | Imputed | 0.18 | 0.22 | 0.18 | 0.17 | 0.31 | 0.35 |
| 6 | rs10947233 | 32124424 | T/G | 0.898 | ncRNA  _intronic | 1.00 | Genotyped | 0.16 | 0.16 | 0.14 | 0.17 | 0.17 | 0.07 |
| 17 | rs146362423 | 21488273 | C/G | 0.509 | intergenic | 0.68 | Imputed | 0.10 | 0.10 | 0.07 | 0.10 | 0.08 | 0.04 |
| 7 | rs1568778 | 109250932 | T/C | 0.824 | intergenic | 0.87 | Imputed | 0.27 | 0.23 | 0.29 | 0.28 | 0.27 | 0.18 |
| 7 | rs115881004 | 68928894 | T/C | 1.000 | intergenic | 0.89 | Imputed | 0.12 | 0.10 | 0.14 | 0.12 | 0.12 | 0.12 |
| 6 | rs114968045 | 32666960 | C/T | 0.584 | intergenic | 0.81 | Imputed | 0.18 | 0.21 | 0.17 | 0.18 | 0.16 | 0.12 |
| 14 | rs11623972 | 73381520 | G/A | 0.817 | intergenic | 0.97 | Imputed | 0.31 | 0.30 | 0.27 | 0.31 | 0.30 | 0.39 |
| 9 | rs10758715 | 5882401 | C/T | 0.804 | intergenic | 0.93 | Imputed | 0.24 | 0.20 | 0.24 | 0.25 | 0.24 | 0.36 |

Chr, chromosome; Alt, alternative allele; Ref, reference allele; HWE, Hardy-Weinberg equilibrium; MAF, minor allele frequency; Kref: Korean reference data

Table S2. Results of the top 10 most significant SNPs after adjusting the effect of smoking.

| Chr | SNP | Position | Alleles | LR | *P*-value* | $\beta^{**}$ | *P*-value** | $\beta^{***}$ | *P*-value*** | $\beta^{****}$ | *P*-value**** | **Gene** |
| --- | --- | --- | --- | --- | --- | --- | --- | --- | --- | --- | --- | --- |
| 5 | rs2961757 | 151936718 | T/G | 36.07 | $1.47\times{10}^{-8}$ | -0.15 | 0.0825 | 0.28 | $9.08\times{10}^{-8}$ | 0.44 | $3.61\times{10}^{-6}$ | *NMUR2, LINC01470* |
| 18 | rs9303897 | 2306842 | G/A | 17.74 | $0.0001$ | -0.48 | $1.28\times{10}^{-5}$ | -0.01 | 0.9157 | 0.47 | $5.87\times{10}^{-5}$ | *LINC00470, METTL4* |
| 6 | rs1770 | 32627833 | G/A | 29.59 | $3.75\times{10}^{-7}$ | -0.16 | 0.1014 | 0.29 | $1.36\times{10}^{-6}$ | 0.46 | $1.19\times{10}^{-5}$ | *HLA-DQB1-AS1* |
| 6 | rs10947233 | 32124424 | T/G | 30.19 | $2.78\times{10}^{-7}$ | -0.28 | 0.0057 | 0.27 | $1.81\times{10}^{-5}$ | 0.54 | $2.93\times{10}^{-7}$ | *PPT2-EGFL8* |
| 17 | rs146362423 | 21488273 | C/G | 19.47 | $5.91\times{10}^{-5}$ | 0.34 | 0.0032 | -0.21 | 0.0068 | -0.54 | $1.47\times{10}^{-5}$ | *C17orf51, UBBP4* |
| 7 | rs1568778 | 109250932 | T/C | 20.86 | $2.96\times{10}^{-5}$ | 0.08 | 0.3451 | -0.23 | $2.88\times{10}^{-5}$ | -0.31 | 0.0007 | *C7orf66, EIF3IP1* |
| 7 | rs115881004 | 68928894 | T/C | 20.82 | $3.01\times{10}^{-5}$ | -0.10 | 0.2426 | -0.25 | $6.92\times{10}^{-6}$ | -0.15 | 0.1086 | *LOC102723427, LOC100507468* |
| 6 | rs114968045 | 32666960 | C/T | 25.16 | $3.44\times{10}^{-6}$ | -0.41 | $7.79\times{10}^{-7}$ | -0.07 | 0.1789 | 0.35 | 0.0001 | *HLA-DQB1, HLA-DQA2* |
| 14 | rs11623972 | 73381520 | G/A | 25.66 | $2.68\times{10}^{-6}$ | -0.65 | $4.79\times{10}^{-6}$ | 0.08 | 0.3134 | 0.73 | $1.11\times{10}^{-6}$ | *DPF3, DCAF4* |
| 9 | rs10758715 | 5882401 | C/T | 27.56 | $1.04\times{10}^{-6}$ | 0.43 | $7.97\times{10}^{-7}$ | -0.05 | 0.3476 | -0.48 | $2.97\times{10}^{-7}$ | *ERMP1, MLANA* |

Chr, chromosome; LRT, likelihood ratio; AIC, Akaike information criterion; *, LR test; **, COPD-Control; ***, Asthma-Control; ****, Asthma-COPD

Table S3. Demographic characteristics of UK Biobank

| Variable | Asthma  (N = 13,475) | COPD  (N = 7,822) | Control  (N = 109,871) | Total  (N = 131,172) |
| --- | --- | --- | --- | --- |
| Age (yr) | 54.6 ± 8.1 | 60.1 ± 6.7 | 56.0 ± 8.0 | 56.1 ± 8.0 |
| Female sex, n (%) | 8,561 (63.5) | 3,424 (43.8) | 63,617 (57.9) | 75,602 (57.6) |
| Height (cm) | 167.1±9.1 | 169.5±9.1 | 168.1±9.0 | 168.1±9.1 |
| BMI | 28.2 ± 5.3 | 27.5 ± 5.0 | 27.4 ± 4.7 | 27.5 ± 4.8 |
| Pre-BD  FEV1 pred | 2.7 ± 0.7 | 1.9 ± 0.6 | 2.8 ± 0.7 | 2.8 ± 0.7 |
| Pre-BD  FEV1/FVC (%) | 75.1 ± 5.5 | 63.0 ± 7.5 | 76.9 ± 5.0 | 75.8 ± 6.2 |

Mean ± SD values are shown for each cell type. BMI, body mass index; FEV1, forced expiratory volume in 1 s; BD, bronchodilator; FVC, forced vital capacity.

Table S4. Replication in UK Biobank

| **Chr** | **Target SNP** | **Correlated SNP (**$\boldsymbol{R}^{\boldsymbol{2}}\boldsymbol{)}$ | **Alt/**  **Ref** | **MAF** | | | **LRT** | ***P*-value*** | $\boldsymbol{\beta}^{\boldsymbol{**}}$ | ***P*-value**** | $\boldsymbol{\beta}^{\boldsymbol{***}}$ | ***P*-value***** | $\boldsymbol{\beta}^{\boldsymbol{****}}$ | ***P*-value****** |
| --- | --- | --- | --- | --- | --- | --- | --- | --- | --- | --- | --- | --- | --- | --- |
|  |  |  |  | **UKB** | | **1000G** |  |  |  |  |  |  |  |  |
| 5 | rs2961757 | rs12655008(0.51) | T/C | 0.46 | 0.51 | | 4.50 | 0.1051 | -0.03 | 0.0486 | 0.01 | 0.5130 | 0.04 | 0.0431 |
| 18 | rs9303897 | rs9955548(0.75) | C/G | 0.32 | 0.36 | | 0.74 | 0.6923 | 0.01 | 0.4600 | -0.01 | 0.6866 | -0.02 | 0.3866 |
| 6 | rs1770 | rs9273508(0.96) | A/G | 0.43 | 0.38 | | 4.66 | 0.0975 | 0.06 | 0.0003 | 0.00 | 0.9348 | -0.06 | 0.0056 |
| 6 | rs10947233 |  | T/G | 0.06 | 0.07 | | 11.22 | 0.0037 | -0.14 | 0.0002 | 0.05 | 0.0489 | 0.21 | $2.34\times{10}^{-6}$ |
| 17 | rs146362423 | rs8075546(0.02) | T/G | 0.42 | 0.44 | | 0.63 | 0.7282 | 0.01 | 0.5998 | 0.01 | 0.5189 | 0.00 | 0.9799 |
| 7 | rs1568778 | rs73194273(0.18) | T/A | 0.02 | 0.01 | | 8.16 | 0.0169 | 0.04 | 0.4969 | 0.12 | 0.0042 | 0.08 | 0.2282 |
| 7 | rs115881004 |  | T/C | 0.14 | 0.12 | | 8.42 | 0.0149 | -0.05 | 0.0409 | -0.04 | 0.0280 | 0.01 | 0.7788 |
| 6 | rs114968045 | rs28891491(0.31) | A/G | 0.23 | 0.27 | | 10.36 | 0.0056 | -0.05 | 0.0180 | 0.05 | 0.0005 | 0.10 | $2.79\times{10}^{-5}$ |
| 14 | rs11623972 | rs12887756(0.57) | G/A | 0.34 | 0.43 | | 3.25 | 0.1968 | 0.03 | 0.0777 | 0.01 | 0.5699 | -0.02 | 0.2775 |
| 9 | rs10758715 | rs12378571(0.35) | T/G | 0.24 | 0.38 | | 1.23 | 0.5400 | 0.01 | 0.4826 | -0.01 | 0.4118 | -0.03 | 0.2702 |

Chr, chromosome; Alt, alternative allele; Ref, reference allele; MAF, minor allele frequency; UKB, UK Biobank; 1000G, 1000 Genome Project; *, LR test; **, COPD-control; ***, asthma-control; ****, asthma-COPD

**Supplementary figures**


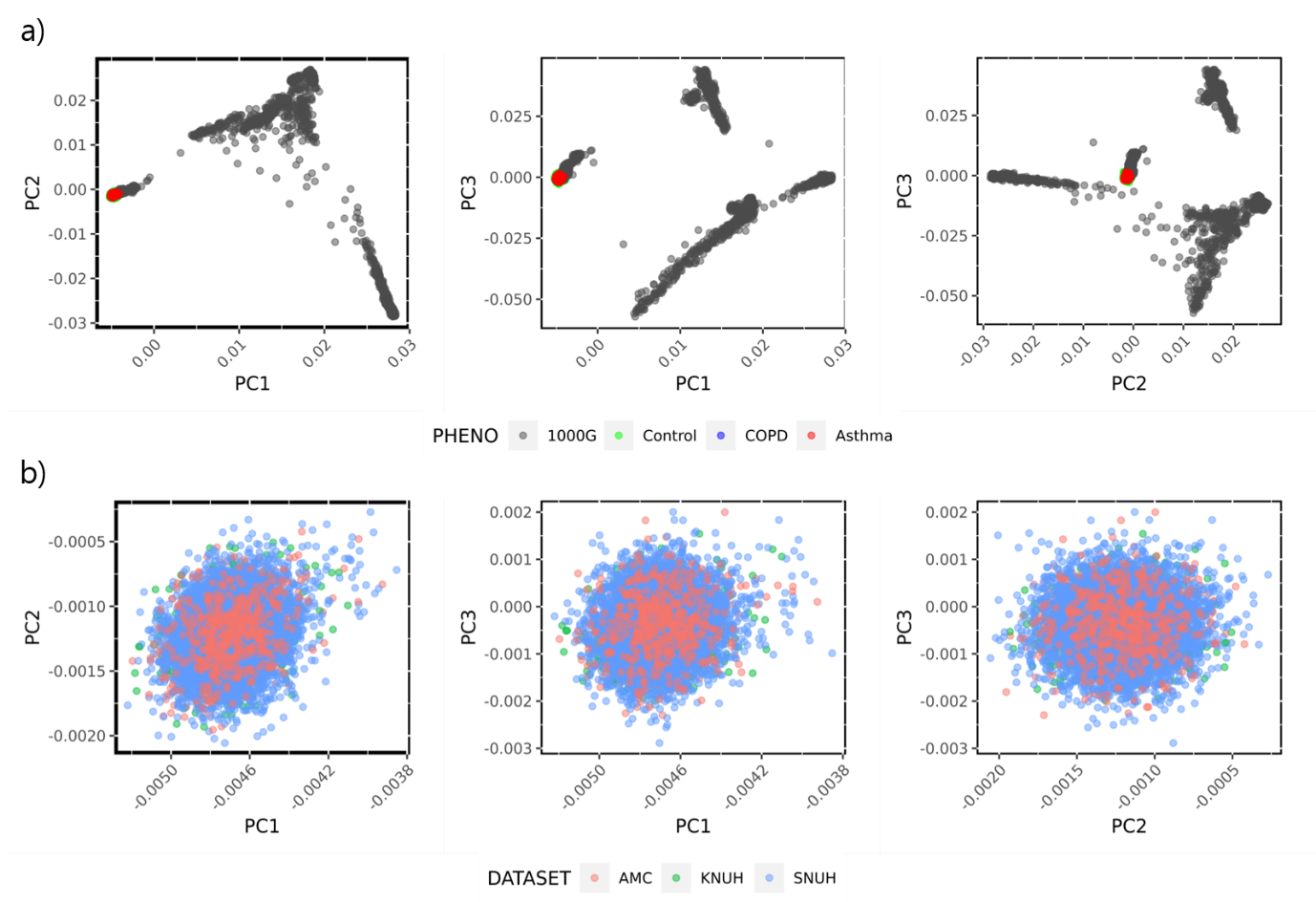


**Figure S1**. Multidimensional scaling (MDS) components were provided. (a) was generated with the discovery dataset and the 1000G dataset. Each subject in the discovery dataset was labeled with their disease status. (b) included only the discovery dataset with better resolution. Each subject was denoted with his/her participating cohort.


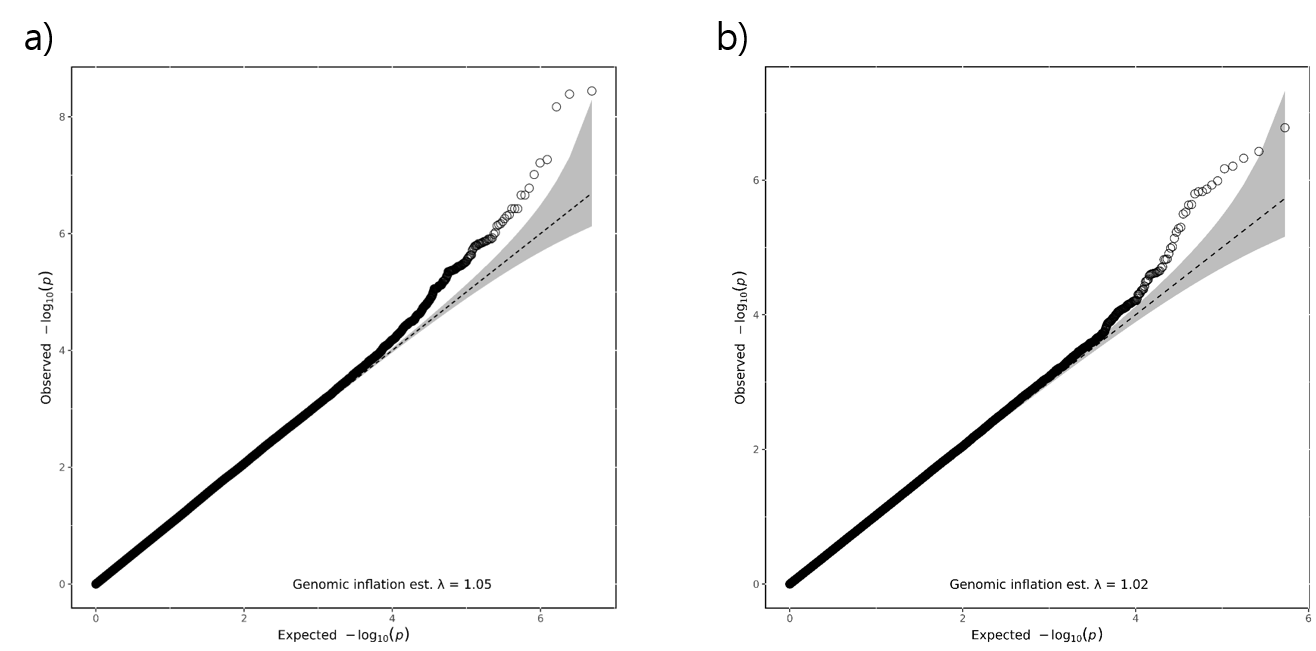


**Figure S2**. Quantile-quantile (QQ) plot for likelihood ratio test (LRT) of discovery dataset. (a) QQ plot for all SNPs, and (b) QQ plot for all pruned SNPs.


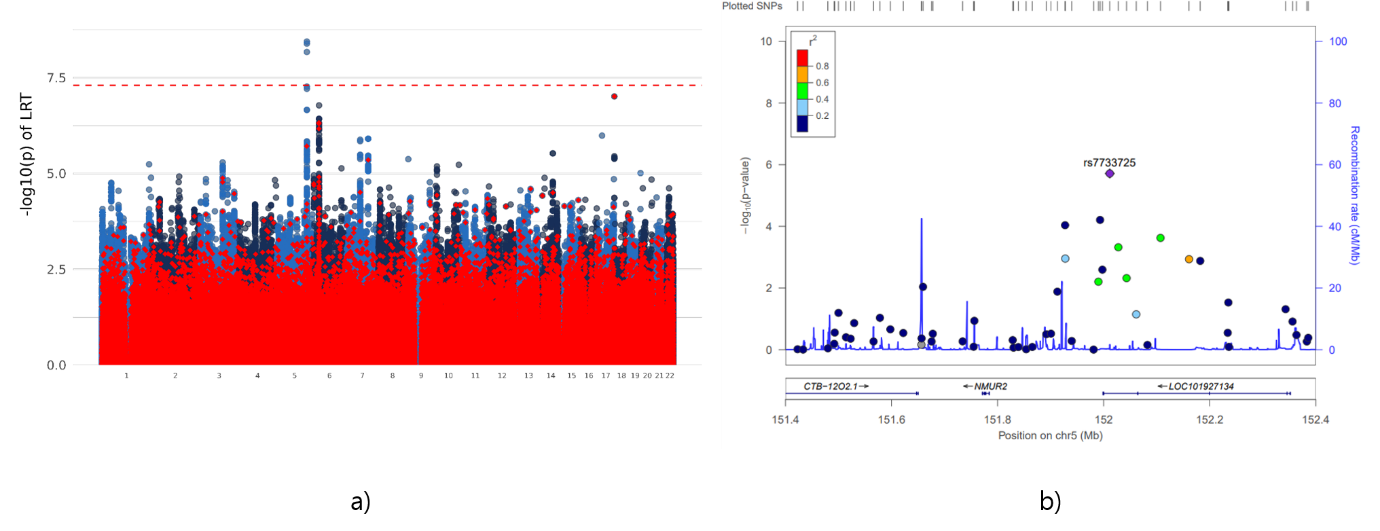


Figure S3. Genome-wide association study (GWAS) results for the Korean dataset using Genotyped SNPs. (a) Manhattan plot of the likelihood ratio test for SNPs from chromosome 1 to 22. Red and blue dots show genotyped and imputed SNP, respectively. (b) An expanded Manhattan plot of the 800 kb region at the genome-wide significant SNP for only genotyped SNPs.


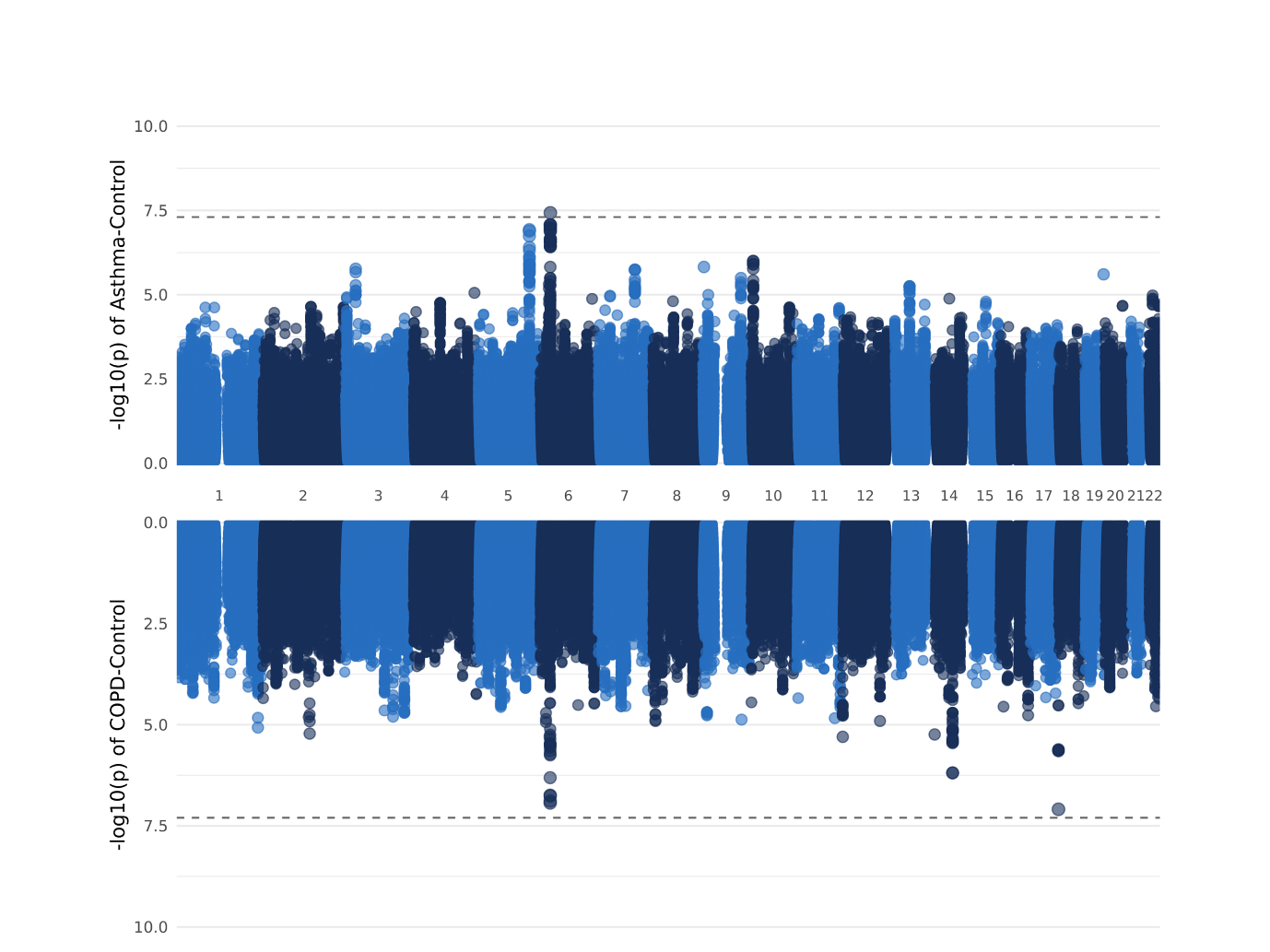


**Figure S4.** Miami plot for the Korean dataset. The Manhattan plot at the top shows the association between asthma and the controls, and the Manhattan plot at the bottom shows COPD and the controls.


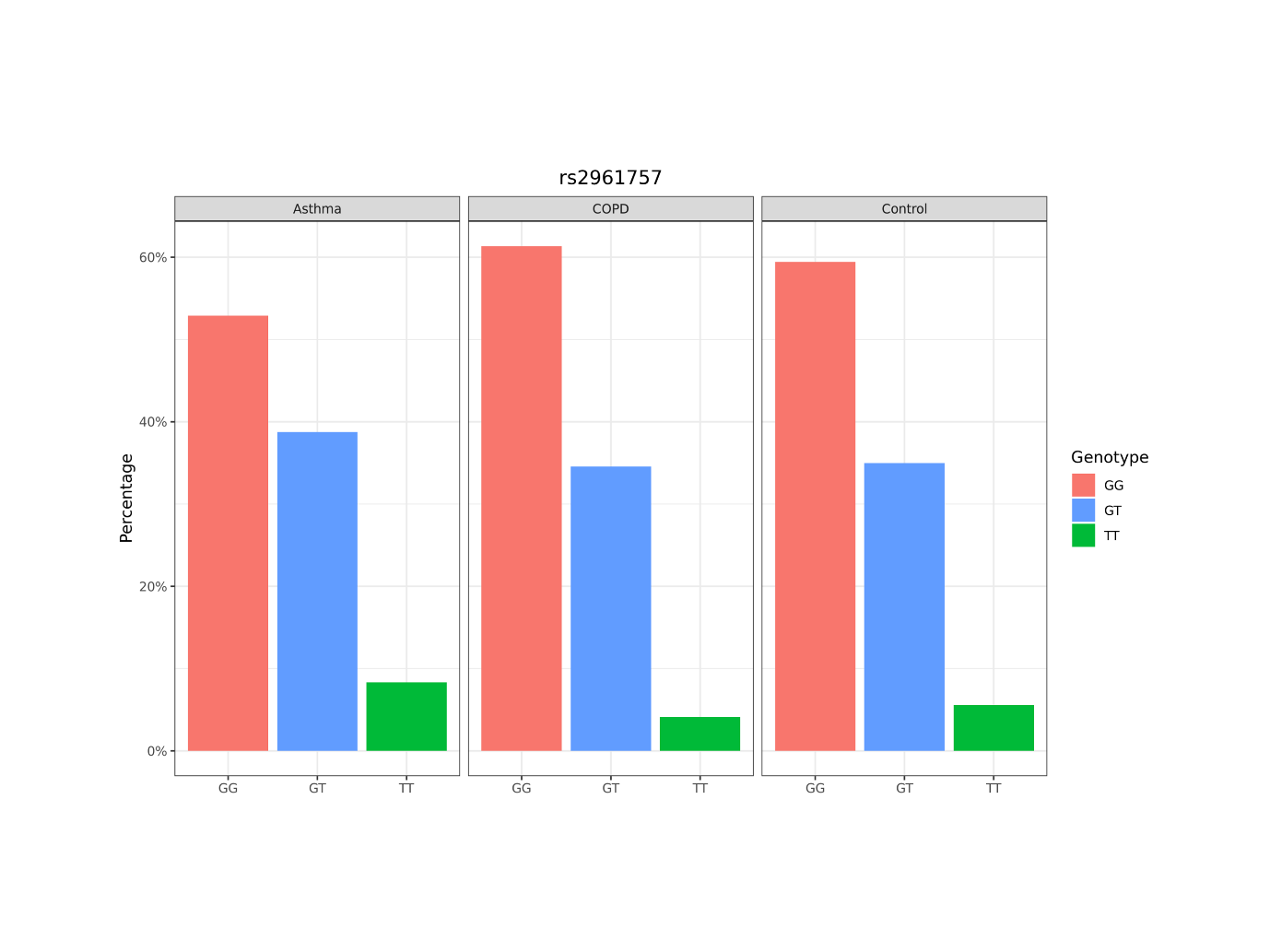
**Figure S5**. Genotype frequencies of rs2961757 in asthma, COPD, and control groups. The red, blue, and green bars represent the proportions of genotypes GG, GT, and TT, respectively, in asthma, COPD, and control groups. The frequency of a minor allele T of rs2961757 in asthma was higher than that in COPD.


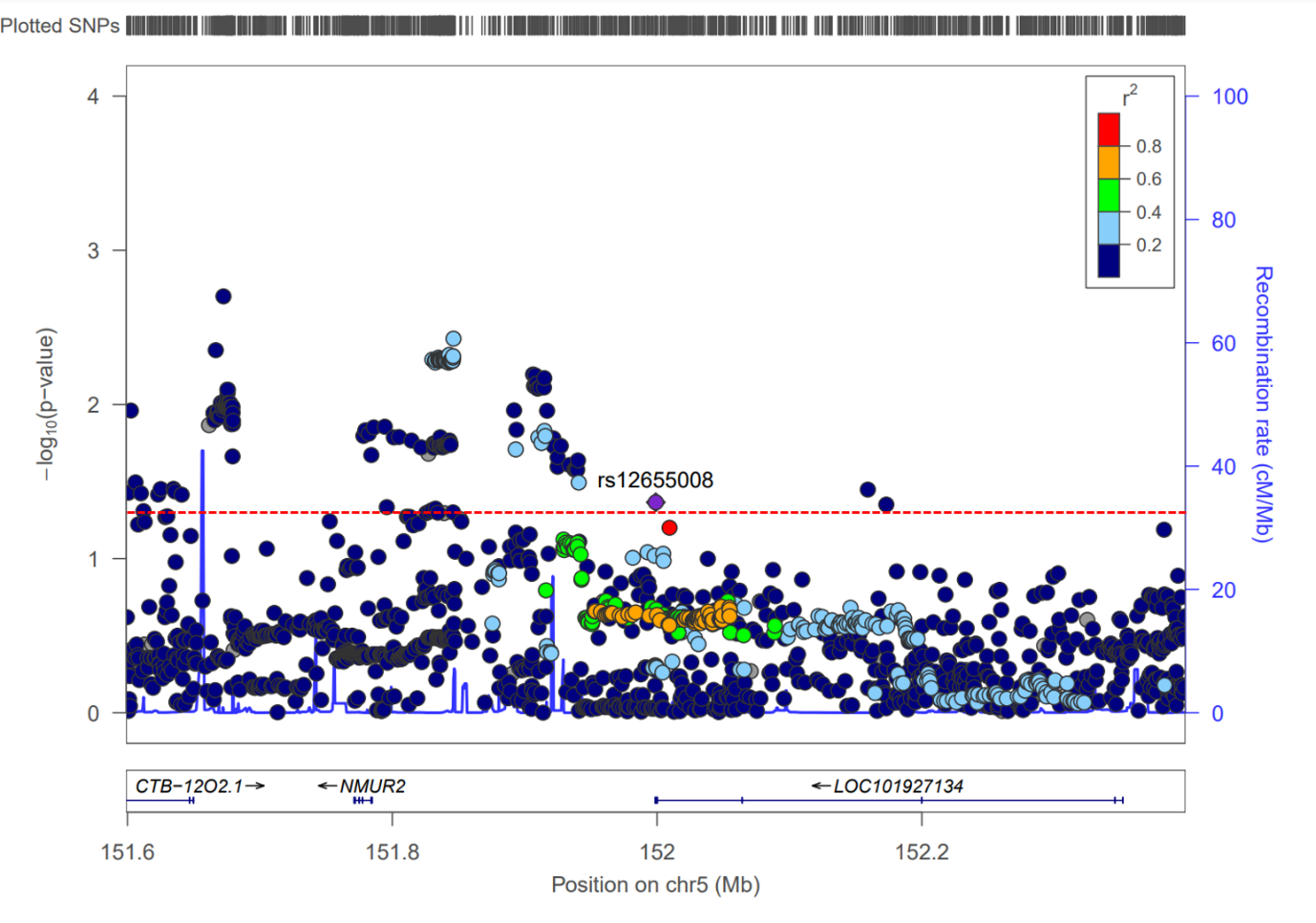


Figure S6. GWAS results for the UK Biobank dataset. An expanded Manhattan plot of the 800 kb region is provided for imputed and genotyped SNPs.


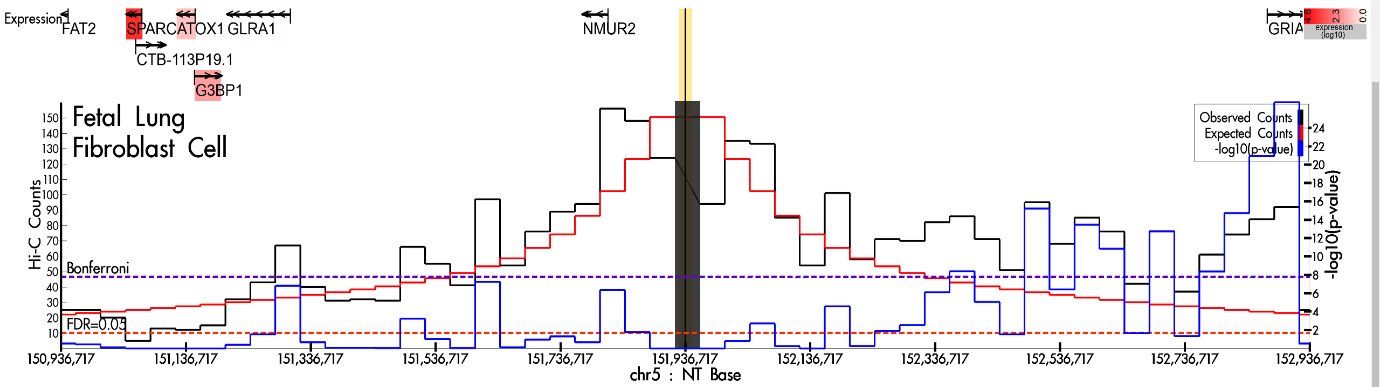


Figure S7. Hi-C count of fetal lung fibroblasts in Chromosome 5.

**Supplementary Method**

***RNA expression association tests***

U-BIOPRED (Unbiased Biomarkers in Prediction of Respiratory Disease Outcomes) is a multicenter prospective cohort study involving 16 clinical centers in 11 European countries. The dataset has four adult groups: (A) severe non-smoking asthma patients, (B) smokers and ex-smokers with severe asthma, (C) mild/moderate non-smoking asthma patients, and (D) healthy non-smoking controls. In this study, we used the mRNA expression of 107 subjects, consisting of 81 asthmatic patients (A, B, and C) and 26 controls (D). Their mRNAs were measured using GPL 570 of Affymetrix using bronchial biopsy. A t-test was performed to identify differentially expressed genes. We also used five gene expression datasets (GSE104472, GSE23552, GSE59019, GSE29133, and GSE112260) downloaded from the GEO database (https://www.ncbi.nlm.nih.gov/geo). GSE104472 consists of 12 asthmatic patients and 12 controls. The mRNA expression of their bronchial epithelia was measured by GPL21185 of Agilent. The GSE23552 dataset included the mRNA expression of Nasosinus tissue from 22 asthmatic patients and 17 controls measured by GPL5175 of Affymetrix. Twenty-six asthmatic patients and 12 controls in GSE59019 were measured by GPL15207 of Affymetrix using peripheral blood mononuclear cell (PBMC). GSE29133 included mRNA expression in lung tissue from three COPD patients and three controls measured by GPL570 of Affymetrix. GSE112260 was measured by GPL22067 of Affymetrix using alveolar macrophages isolated from eight asthmatic and four COPD patients. All GEO datasets were normalized with a 2% trimmed mean and analyzed using Limma after adjusting for sex and age. For GSE59019, stimulation (low-molecular-weight hyaluronan, LMWHA) was available and included as an additional covariate.
